# CRISPR-Cas13a Targeting the Enhancer RNA-SMAD7e Inhibits Bladder Cancer Development Both *in vitro* and *in vivo*

**DOI:** 10.3389/fmolb.2020.607740

**Published:** 2020-11-17

**Authors:** Wenan Che, Shanting Ye, Aoxiang Cai, Xiaojuan Cui, Yuandong Sun

**Affiliations:** ^1^Hunan Key Laboratory of Economic Crops Genetic Improvement and Integrated Utilization, School of Life Sciences, Hunan University of Science and Technology, Xiangtan, China; ^2^Shenzhen Second People’s Hospital, The First Affiliated Hospital of Shenzhen University, Shenzhen, China

**Keywords:** CRISPR-Cas13a, SMAD7e, bladder cancer, estrogen, enhancer RNA (eRNA)

## Abstract

Enhancers are cis-acting elements that can promote the expression of target genes and respond to estrogen to induce the transcription of eRNAs, which are closely associated with cancer development. Further study on eRNAs may lead to a better understanding of the significance of transcriptional regulation and the progression of malignant tumors. SMAD7 enhancer RNA (SMAD7e) is an estrogen-responsive eRNA. However, the relationship between SMAD7e and bladder cancer remains unclear. SMAD7e was significantly upregulated in bladder cancer tissues and estrogen-stimulated cells. Knockdown of SMAD7e by CRISPR-Cas13a suppressed cell proliferation and migration, and induced cell apoptosis and inhibited cell invasion. Estrogen caused overexpression of SMAD7e and played a facilitating role in bladder cancer cells. Furthermore, knockdown of SMAD7e by CRISPR-Cas13a prevented the cancer-promoting effects of estrogen on bladder cancer both *in vitro* and *in vivo*. The present study suggested the crucial role of SMAD7e in bladder cancer. Estrogen might promote the development of bladder cancer by inducing SMAD7e production. These findings may provide a potential target for CRISPR-mediated gene therapy for bladder cancer in the future.

## Introduction

Estrogen is reported to be associated with the development of many cancers (Horie, 2010). To the best of our knowledge, bladder cancer mainly occurs in males, while female patients have lower survival rates (Hartge et al., 1990; Madeb and Messing, 2004; Xu et al., 2013; Murakawa et al., 2016). The sex difference in morbidity suggests the importance of estrogen for bladder cancer development. However, the molecular mechanisms of estrogen in bladder cancer remain unclear. Although there are many treatments for patients with bladder cancer, such as chemotherapy, radiation, and surgery, the 5-year survival rate is low (Racioppi et al., 2012; Sofra et al., 2013; Rose and Milowsky, 2016). Therefore, there is a need to develop a more effective and safer method for treating bladder cancer.

Recently, several studies suggested that natural enhancers were occupied by RNA polymerase II (RNAP II) and transcribed into non-coding (nc) RNAs termed enhancer RNAs (eRNAs) (De Santa et al., 2010; Kim et al., 2010). The eRNA-producing enhancer regions have been exploited and eRNAs may play a crucial role in gene transcriptional regulation (Lam et al., 2013). Studies have confirmed that the abnormal expressions of eRNAs are closely related to diseases (He et al., 2013; IIott et al., 2014; Yao et al., 2015; Bal et al., 2017; Gras et al., 2017). SMAD7 is proved to be an intracellular protein with a well-known ability to antagonize transforming growth factor-β1 (TGF-β1) signaling through multiple mechanisms (Nakao et al., 1997). Recent studies have shown that SMAD7 plays a role in breast cancer development and progression (Rahman et al., 2017). SMAD7 facilitated the proliferation of cancer cells originating from colorectal, pancreatic, prostate, and lung (Brionesorta et al., 2011). Previous studies found that the corresponding enhancer of SMAD7 could be transcribed into functional transcripts – SMAD7e with estrogenic stimulation (Hah et al., 2013; Li et al., 2013). However, the roles of SMAD7e in bladder cancer are completely unclear. It would therefore be interesting to determine if the SMAD7e mediates the effect of estrogen on bladder cancer.

In this study, we examined the clinical significance of SMAD7e in 38 bladder cancer samples. The effects of SMAD7e knockdown on the proliferation, migration, apoptosis, and invasion of bladder cancer cells were determined. Moreover, we determined the promoting effect of estrogen on bladder cancer and the potential molecular mechanisms of estrogen in bladder cancer. In addition, we demonstrated that SMAD7e knockdown mediated by CRISPR-Cas13a reduced the cancer-promoting ability of estrogen on bladder cancer cells both *in vitro* and *in vivo*. This work suggested CRISPR-Cas13a as an effective tool to target one specific enhancer RNA- SMAD7e in bladder cancer and revealed its advantage in anticancer research.

## Results

### SMAD7e Was Overexpressed in Bladder Cancer Tissues and Positively Correlated With Clinicopathological Features

**FIGURE 1:**
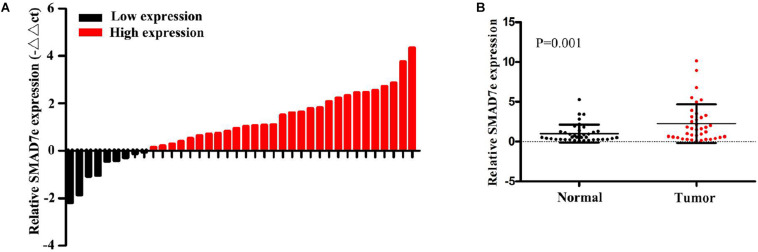
The expression of SMAD7e in bladder cancer. **(A)** SMAD7e was up-regulated in bladder cancer. The relative expression levels of SMAD7e were detected by qPCR in 38 bladder cancer patients. **(B)** The relative expression level of SMAD7e was significantly increased in bladder cancer tissues compared to paired normal tissues. Data are shown as the mean ± SD.

We measured the relative expression levels of SMAD7e in a total of 38 patients with bladder cancer by real-time qPCR. Compared with normal counterparts, the SMAD7e expression was obviously overexpressed in 29 cancer tissues (Figures 1A,B). We further analyzed the relationship between SMAD7e expression and clinical features of patients. As shown in Table 1, there was a positive correlation between the SMAD7e expression level and the clinical features including histological grade and TNM stage of bladder cancer. However, sex and age had no relationship with the SMAD7e expression level. Therefore, SMAD7e may function as a oncogenic factor in bladder cancer.

**TABLE 1:** Correlation between SMAD7e expression and clinicopathological characteristics of bladder cancer patients.

**Characteristics**	**Total**	**SMAD7e expression**		***P* value**
		**Low**	**High**	
**Gender**				
Female	7	3 (28.6%)	4 (71.4%)	0.186
Male	31	6 (32.3%)	25 (67.7%)	
**Age**				
≤60	10	4 (30.0%)	6 (70.0%)	0.157
>60	28	5 (17.9%)	23 (82.1%)	
**Histological grade**				
Low	14	7 (60.0%)	7 (40.0%)	0.004**
High	24	2 (21.7%)	22 (78.3%)	
**TNM stage**				
0/I	6	4 (83.3%)	2 (16.7%)	0.007**
II/III/IV	32	5 (18.8%)	27 (81.2%)	

*^∗∗^*p* < 0.01 was considered significant.*

### Estrogen Induced the Production of SMAD7e, Facilitated Cell Proliferation, Increased Cell Migration, Suppressed Cell Apoptosis, and Promoted Cell Invasion in Bladder Cancer

**FIGURE 2:**
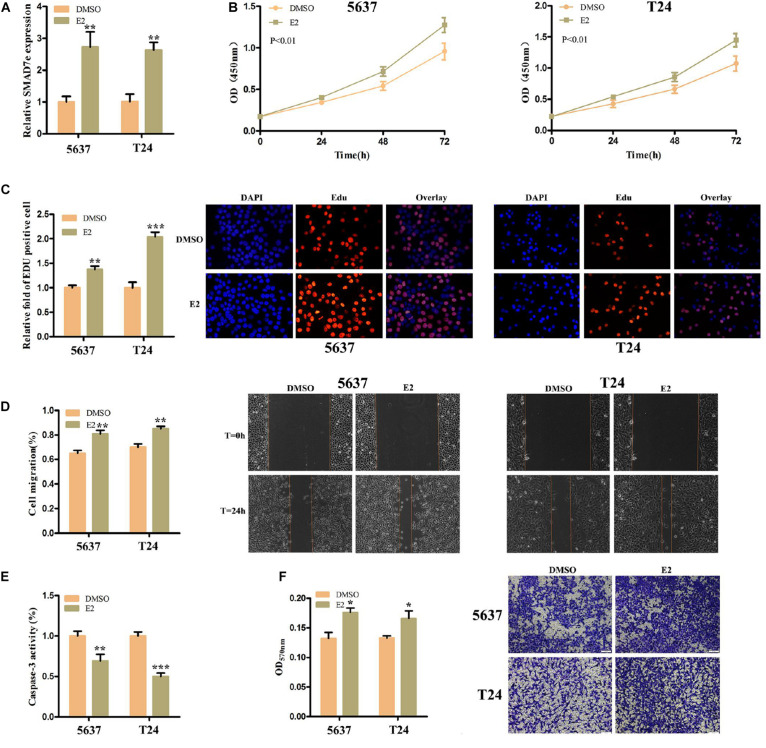
The effect of E2 on bladder cancer cells. **(A)** After stimulation with estrogen for 1 h, the relative expression levels of SMAD7e were significantly increased in bladder cancer cells. **(B,C)** Estrogen facilitated cell proliferation as revealed by CCK8 and Edu assays in 5637 and T24 cells. **(D)** Estrogen promoted cell migration as revealed by wound healing assays in 5637 and T24 cells. **(E)** Estrogen reduced bladder cancer cell apoptosis. **(F)** Estrogen increased cell invasion as detected by Transwell^®^ assays. E2 represents estrogen. Data are shown as the mean ± SD (*p < 0.05, **p < 0.01, ***p < 0.001).

We measured SMAD7e expression levels after estrogen stimulation for 1 h in 5637 cells and T24 cells, using RT qPCR. These results indicated that SMAD7e expression was significantly increased in bladder cancer cells when stimulated by estrogen (Figure 2A). To determine the impact of estrogen on bladder cancer cells, the cell proliferations of 5637 and T24 were determined by CCK8 and Edu assays. Estrogen-promoted cell growth was observed in 5637 cells and T24 cells (Figures 2B,C). We further determined that estrogen promoted cell migration in bladder cancer using wound healing assays (Figure 2D). Furthermore, estrogen suppressed cell apoptosis in bladder cancer. The relative activity of caspase-3 was determined using a caspase 3 ELISA (Figure 2E). Estrogen promoted cell invasion in bladder cancer (Figure 2F). These results demonstrated that estrogen played a cancer-promoting role in bladder cancer.

### Knockdown of SMAD7e by CRISPR-Cas13a Suppressed Proliferation, Inhibited Migration, Promoted Apoptosis, and Decreased Invasion in Bladder Cancer Cells

**FIGURE 3:**
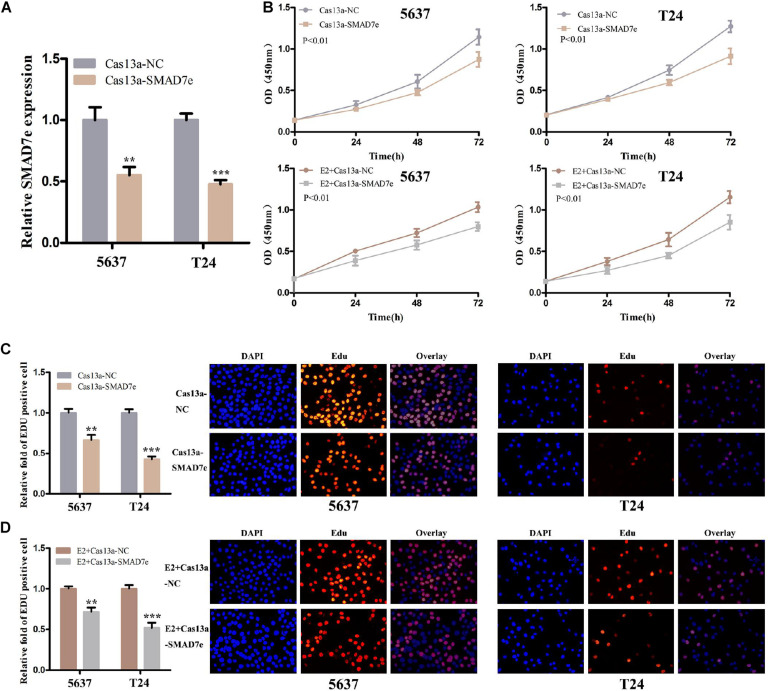
The effect of Cas13a-SMAD7e on the proliferation of E2-treated bladder cancer cells. **(A)** SMAD7e expression levels were dramatically downregulated by Cas13a-SMAD7e. **(B)** Cell proliferation was detected by CCK8 assays in T24 and 5637 cells after transfection for 24 h. Knockdown of SMAD7e by Cas13a inhibited cell growth and reduced the proliferative effects of estrogen as determined by CCK8 assays in 5637 and T24 cells. **(C)** Edu assays were used to measure cell proliferation. Downregulation of SMAD7e inhibited cell growth of bladder cancer cells. **(D)** SMAD7e knockdown reduced the pro-proliferative effects of estrogen in 5637 and T24 cells. E2 represents estrogen. Data are shown as the mean ± SD (**p < 0.01, ***p < 0.001).

**FIGURE 4:**
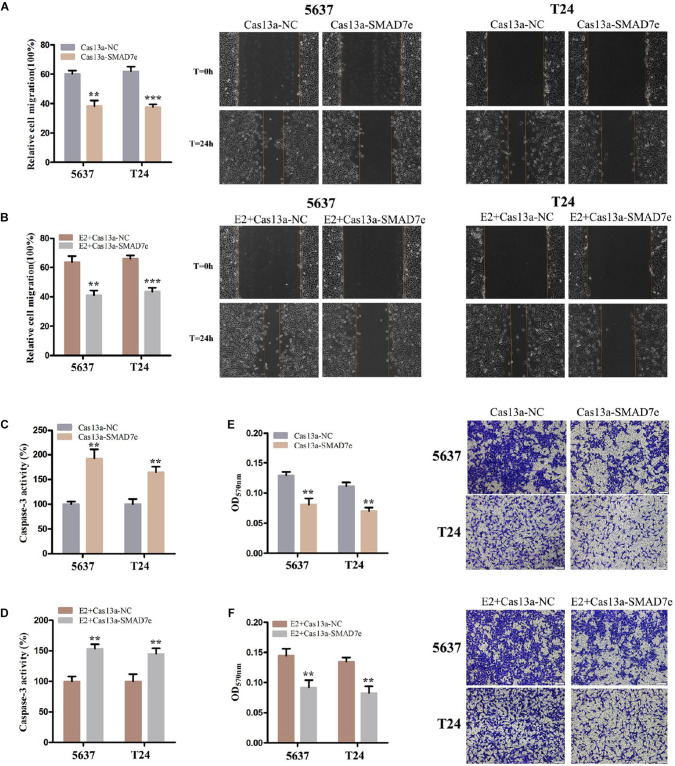
The effect of Cas13a-SMAD7e on the migration of E2-treated bladder cancer cells. **(A,B)** Cell migration was detected by wound healing assays. SMAD7e knockdown inhibited migration of bladder cancer cells induced by estrogen. **(C,D)** The effects of knockdown of SMAD7e and SMAD7e knockdown with estrogen treatment were measured by caspase 3 ELISA assays. **(E,F)** Knockdown of SMAD7e and downregulation of SMAD7e attenuated estrogen-induced invasion of bladder cancer cells. E2 represents estrogen. Data are shown as the mean ± SD (**p < 0.01, ***p < 0.00).

CRISPR-Cas13a was used to measure the effect of SMAD7e knockdown on biological behaviors of bladder cancer cells. After Cas13a-SMAD7e or Cas13a-NC transfection for 24 h, the relative expression levels of SMAD7e were significantly reduced (Figure 3A). We used CCK8 and Edu assays to compare the cell proliferations between the SMAD7e knockdown group and the negative control group. The results showed that knockdown of SMAD7e suppressed cell growth in 5637 and T24 cells (Figures 3B,C). The cell migration was significantly inhibited, as shown by wound healing assays in 5637 and T24 cells (Figure 4A). We also used caspase 3 ELISA assays to compare the cell apoptosis rates of the two groups. The results indicated that knockdown of SMAD7e increased cell apoptosis (Figure 4C). Furthermore, knockdown of SMAD7e decreased cell invasion, as demonstrated using Transwell^®^ assays (Figure 4E). Therefore, knockdown of SMAD7e inhibited the tumorigenicity of bladder cancer cells.

### Knockdown of SMAD7e Attenuated the Carcinogenesis Effect of Estrogen in Bladder Cancer Cells

We hypothesized SMAD7e played a key role in the carcinogenic effects of estrogen in bladder cancer. Bladder cancer cells were transfected with Cas13a-SMAD7e or Cas13a-NC vectors and treated with estrogen. Compared with the negative control, cell proliferation was obviously decreased (Figures 3B,D); the cell migration ratio was reduced (Figure 4B); the cell apoptosis was increased (Figure 4D); and the invasive ability of the cells was also prominently weakened (Figure 4F) in the SMAD7e knockdown group treated with estrogen.

**FIGURE 5:**
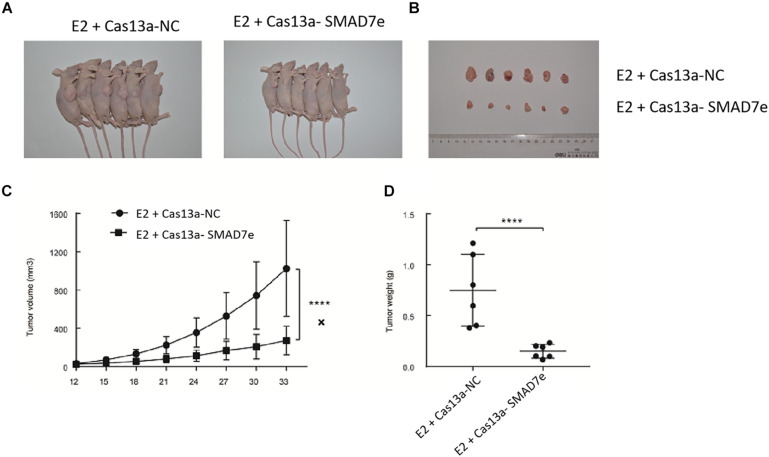
The effect of Cas13a-SMAD7e on the *in vivo* growth of E2-treated bladder cancer cells. **(A)** Representative images of xenograft models. **(B)** Xenograft tumors from respective groups were shown after injection with SMAD7e stable knockdown T24 cells and vector transfected T24 cells. **(C)** Tumor growth curves were measured every 3 days. **(D)** Average weight of excised tumors. ****p < 0.0001.

To verify the effects of SMAD7e on the carcinogenic effects of estrogen *in vivo*, xenograft models were established by injecting stable knockdown SMAD7e T24 cells and vector transfected T24 cells into subcutaneous tissues of nude mice. All nude mice developed xenogeneic tumors at the injection site (Figure 5A). Tumor growth of SMAD7e silenced cells was slower than that of the SMAD7e knockdown group treated with estrogen (Figure 5B). As shown in Figures 5C,D, downregulation of SMAD7e significantly decreased the xenograft tumor volume and tumor weight compared to the SMAD7e knockdown group treated with estrogen. In conclusion, knockdown of SMAD7 attenuated the effects of estrogen on bladder cancer.

## Discussion

Previous studies on gene-related cancers have focused on coding genes, because they directly affect cell function. However, accumulating evidences has indicated that ncRNAs play a non-redundant role in regulating gene transcription and protein generation, including the eRNAs (Murakawa et al., 2016). Compared to other mRNAs and lncRNAs, eRNAs can promote the development of various cancers by regulating the expression of multiple genes, and should be ideal anticancer targets.

It has been reported that human breast cancer cell E2 binding to estrogen receptor α leads to increased transcription of eRNAs on enhancers, along with the upregulation of E2-associated coding genes (Wang et al., 2011). The corresponding enhancers of SMAD7 can respond to estrogen stimuli to transcribe SMAD7 eRNA. The eRNAs appear rapidly after induction, and the half-life is very short. The mean transcription unit length of eRNAs from the estrogen receptor binding site is ∼3–5 kb, and therefore eRNAs may interact with enhancers.

The production of eRNAs is positively correlated with the expression of the target genes. Increased evidence suggests that eRNAs may affect cell biological behaviors, such as cell migration, invasion, proliferation, and apoptosis by upregulating the expression of target genes. Some studies further indicates that CREB-binding protein (CBP) can directly bind to eRNAs (Castello et al., 2012). CBP controls gene expression patterns in organisms by regulating histone acetylation, reducing histone affinity for DNA, and facilitating chromatin loosening to promote transcription. Although some studies have shown that eRNAs play a role in gene regulation, further studies are needed to reveal their biological functions.

SMAD7e is an estrogen-associated eRNA that enhances gene transcription. Our study was the first to demonstrate the overexpression of SMAD7e in bladder cancer tissues. Further research demonstrated that the expression level of SMAD7e was closely associated with the histological grade and the TNM stage. Our results suggested that SMAD7e may contribute to the initiation and progression of bladder cancer. In addition, we demonstrated that estrogen could induced the synthesis of SMAD7e and promoted the malignant biological behaviors of bladder cancer cells. Therefore, SMAD7e is a potential anticancer target in human bladder cancer.

To further verify the function of SMAD7e in bladder cancer, we knocked down SMAD7e by CRISPR-Cas13a (Abudayyeh et al., 2017). Since there lacks an effective tool to target this specific type of non-coding RNA, we therefore used the newly developed biological method- CRISPR-Cas13a to resolve the targeting problem. Some recent studies (Zhao et al., 2018; Qi et al., 2019; Chen et al., 2020) also used CRISPR-Cas13a to inhibit different cancers by suppressing oncogenic mRNAs. Our results showed that downregulation of SMAD7e suppressed proliferation and migration, promoted apoptosis, and decreased invasion in bladder cancer. Therefore, it was suggested that SMAD7e had a cancer-promoting effect in bladder cancer. We also hypothesize that there might be a unique relationship between SMAD7e and the tumor-promoting action of estrogen, whereby estrogen causes the induction of SMAD7e. Lastly, we observed the cell malignant abilities after estrogen treatment was impaired after Cas13a-SMAD7e transfection both *in vitro* and *in vivo*. These results demonstrated that SMAD7e might contribute to the carcinogenic effects of estrogen on bladder cancer, and that Cas13a-SMAD7e should be a powerful molecular approach to inhibit bladder cancer development.

## Materials and Methods

### Patients and Tissue Specimens

Thirty-eight bladder urothelial carcinoma patients who received radical cystectomy were included in this study. The samples were stored in liquid nitrogen immediately after resection. These methods were based on approved guidelines. Formal written approvals from these patients were also received. The study was approved by the Research Ethics Committee of our institute.

### Cell Culture and Treatments

Human bladder cancer cell lines (5637, T24) were obtained from the America Type Culture Collection (ATCC, Manassas, VA, United States). The 5637 cells were grown in RPMI-1640 medium (Gibco BRL, Gaithersburg, MD, United States) supplemented with 10% charcoal-stripped fetal bovine serum (FBS; Gibco BRL) and 1% antibiotics (100 U/mL penicillin and 100 μg/mL streptomycin sulfates) in a 5% CO_2_ humidified incubator at 37°C. The T24 cells were cultured in DMEM medium (Gibco BRL), supplemented with 10% charcoal-stripped FBS (Gibco BRL) and 1% antibiotics (100 U/mL penicillin and 100 μg/mL streptomycin sulfates) in a 5% CO_2_ humidified incubator at 37°C. Cells were treated with 10 nM DMSO (Sigma-Aldrich, St. Louis, MO, United States) or 10 nM estrogen (Sigma-Aldrich) for 1 h to induce SMAD7e.

### CRISPR-Cas13a Vector Transfections

CRISPR-Cas13a targeting SMAD7e (Cas13a-SMAD7) and negative control (Cas13a-NC) were purchased from Syngen Tech Co., Beijing, China. Bladder cancer cells 5637 and T24 were seeded in 6-well plates and were transfected with 2.5 μg Cas13a-SMAD7e or Cas13a-NC when 80–90% confluent.

### RNA Extraction and Real-Time qPCR Analysis

The total RNA of the tissue samples were extracted using the TRIzol reagent (Invitrogen, Carlsbad, CA, United States) according to manufacturer’s instructions. The cDNA was synthesized using a Revertra Ace qPCR RT Kit (Toyobo, Osaka, Japan) according to the instructions. qPCR was carried out using real time PCR Master Mix (Toyobo) according to the instructions under conditions of 40 cycles of 15 s at 95°C, 15 s at 60°C, and 45 s at 75°C using the ABI PRISM 7500 Fluorescent Quantitative PCR System (Applied Biosystems, Foster City, CA, United States). The 2^–ΔΔCt^ method was chosen to calculate the relative expression levels.

### Cell Proliferation Assays

Cell proliferation was determined using a Cell Counting Kit-8 (CCK-8) (TransGen, Beijing, China) and 5-ethynyl-20-deoxyuridine (Edu) assay kit (Ribobio, Guangzhou, China) China according to the instructions. For CCK-8 assays, cells were incubated in a 96-well plate for 24 h and then transiently transfected with CRISPR-Cas13a. The absorbance was detected at 0, 24, 48, and 72 h after transfection by a microplate reader (Bio-Rad, Hercules, CA, United States). Each test was carried out at least three times.

### Cell Migration Assays

Cell migration was detected using the wound healing assay. Bladder cancer 5637 and T24 cells were cultured and transfected with either Cas13a-SMAD7e or Cas13a-NC for 24 h. For the wound healing assay, a sterile 200 μL pipette tip was used to create clear lines and photographs were immediately taken. The cells were cultured in medium supplemented with 1% FBS. The migration distance was observed after 24 h of wound formation and measured by the HMIAS-2000 software program. Experiments were repeated at least three times.

### Cell Apoptosis Assays

Cell apoptosis was determined using enzyme-linked immunosorbent assays (ELISAs). Bladder cancer 5637 and T24 cells were transfected with Cas13a vector in petri plates. After 24 h, the caspase 3 ELISA assay kit (Hcusabio, Wuhan, China) was used to detect the activity of caspase 3 according to the manufacturer’s protocol. OD values were measured at 450 nm by using a microplate reader (Bio-Rad). Each test was carried out at least three times.

### Cell Invasion Assays

Cell invasion was detected using Transwell^®^ assays. For the assay, about 1.5 × 10^5^ 5637 cells and 5 × 10^4^T24 cells supplemented with 200 μL serum-free medium were plated into the upper chambers (24-well insert, pore size 8 μm: Corning, Sunnyvale, CA, United States) containing Matrigel^®^ (1:8, 50 μL/well: BD Bioscience, San Jose, CA, United States). The lower chamber was filled with 500 μL of complete medium with 10% FBS and 1% antibiotics. Cells were cultured for 24 h and then cells under the surface of the lower chamber were washed with 1 × phosphate-buffered saline, fixed with methanol for 20 min, stained with 0.1% Crystal Violet for 25 min, and washed three times. Cells were observed using an inverted microscope and imaged. Each chamber with the invaded cells was than soaked in 1 mL 33% acetic acid for 10 min to wash out the Crystal Violet and 100 μL of 33% acetic acid was added into each well of the 96-well plates. The absorbance was determined at a wavelength of 570 nm using a microplate reader (Bio-Rad). Experiments were repeated at least three times.

### Xenograft Tumor Model

Male BALB/c nude mice (4–6 weeks old), weighting 18–20 g, were purchased from Shanghai Experimental Animal Center (Shanghai, China). All mice were kept in a strict pathogen-free conditions. The Ethics Committee for Animal Experiments of University approved the animal experiments. To establish the xenograft model, a total of 4 × 10^6^ tumor cells were subcutaneously injected into the right flank of the nude mice. Every 3 days, we measured the tumor length and width with caliper. At the end point, the mice were euthanized, and tumor tissues were weighted.

### Statistical Analysis

Each experiment was performed in triplicate. The data are presented as the mean ± standard deviation (SD), and all statistical analyses were conducted using SPSS 17 software (IBM, Chicago, IL, United States). The SMAD7e expression differences between bladder cancer tissues and matched normal tissues were analyzed using paired samples *t*-tests. CCK-8 assay data were analyzed by analysis of variance. Other data were analyzed by the independent samples *t*-test. *P* < 0.05 was regarded as statistically significant.

## Data Availability Statement

The original contributions presented in the study are included in the article/supplementary material, further inquiries can be directed to the corresponding author.

## Ethics Statement

The animal study was reviewed and approved by Research Ethics Committee of Hunan University of Science and Technology.

## Author Contributions

AC, SY, WC, and XC performed the experiments and conducted the data analyses. YS supervised the project and wrote the manuscript. All authors contributed to the article and approved the submitted version.

## Conflict of Interest

The authors declare that the research was conducted in the absence of any commercial or financial relationships that could be construed as a potential conflict of interest.
